# A study on the brain mechanisms of postural control improvement in obese college students through traditional martial arts Tan Tui practice

**DOI:** 10.3389/fpsyg.2025.1681295

**Published:** 2025-10-09

**Authors:** Youhua Li, Quanwei Tang, Qiao Huang

**Affiliations:** ^1^Department of Physical Education, Southeast University, Nanjing, Jiangsu, China; ^2^Sports Department, Wenzhou Vocational College of Science and Technology, Wenzhou, Zhejiang, China

**Keywords:** traditional martial arts, postural control, brain function, obese college students, EEG

## Abstract

**Objective:**

To investigate the effects of traditional martial arts Tan Tui practice on the brain mechanisms of postural control in obese college students.

**Methods:**

Eighty obese college students were randomly divided into a Tan Tui practice group (TT) and a control group (CON), with 40 participants in each group. The TT group engaged in Tan Tui practice, while the CON group performed jogging. The intervention period was 14 weeks (3 times per week, 60 min each session). Absolute power of the alpha frequency band was assessed at baseline (pre-intervention) and after 14 weeks (post-intervention) for brain regions related to cognitive function and sensorimotor function.

**Results:**

(1) Absolute Brain Power: Compared to the control group, the experimental group showed significant differences (*p* < 0.05) in alpha band power in brain regions associated with cognitive function (prefrontal: FP1, FPz, FP2; temporal: T8, P7; occipital: O1) and sensorimotor function (posterior frontal: F7, F3, FZ; parietal: P3, P4; central: C3, CZ). Compared to pre-intervention, the experimental group showed significant differences (*p* < 0.05) in alpha band power after training in cognitive function (prefrontal: FPz; temporal: T7, T8, P7, P8; occipital: O1) and sensorimotor function (posterior frontal, parietal, and central lobes). (2) Brain Region Symmetry: Compared to the control group, the difference in alpha band power between the left and right hemispheres of the occipital lobe (cognitive function) and central lobe (sensorimotor function) showed significant differences (*p* < 0.05) after training. Compared to pre-intervention, the experimental group showed significant differences (*p* < 0.05) in alpha band power after training in sensorimotor function (posterior frontal and central lobes). (3) Brain Region Synergy: Compared to pre-intervention, the experimental group showed a significant positive correlation (*p* < 0.05) in the synergy between the prefrontal and occipital lobes, and the temporal and occipital lobes, which are cognitive function brain regions. After training, there was a significant positive correlation (*P* < 0.05) in the synergy among sensorimotor function brain regions. Furthermore, after training, there was a significant positive correlation (*p* < 0.05) in the synergy between different sensorimotor function brain regions (posterior frontal, parietal, central) and cognitive function-related brain regions.

**Conclusion:**

A 14-week systematic traditional martial arts Tan Tui training program can significantly enhance cortical excitability, improve functional symmetry between the left and right brain hemispheres, and strengthen the synergy among multiple brain regions in obese college students. Our findings demonstrate that traditional martial arts practice induces measurable neural plasticity and improves postural control and brain function in populations. This study links Tan Tui to modern neurophysiological evidence, offering novel insights into mind–body interventions for brain health in obesity.

## 1 Introduction

Obesity among college students has emerged as a growing global public health concern, associated with lifestyle changes (de Vos et al., 2015), academic stress (Hariyanto et al., 2025), and dietary imbalance (Choi, 2020). The overweight and obesity rates for young people aged 18–29 in China have reached 24.7 and 10.9%, respectively, making college students one of the high-risk groups for obesity (Jiang et al., 2018). International studies have found that the obesity rate for American college students is approximately 18.5% (Odlaug et al., 2015). Although the absolute prevalence of obesity in Chinese college students is still lower than in developed countries, longitudinal multicenter studies have shown a significantly faster growth trajectory, underscoring the potential health risks and the increasing prominence of movement disorders and postural control problems related to obesity (Hong et al., 2023).

Obesity leads to a shift in the body's center of gravity and increased load on the lower limb joints, which results in a decline in static balance ability, deficiencies in dynamic balance, and an increased risk of falls. Studies have found that obese college students perform poorly in single-leg standing and dynamic balance tests, with significantly greater sway compared to their normal-weight peers (Andreato et al., 2020; do Nascimento et al., 2017; Huang et al., 2024). Excess abdominal adiposity may mechanically restrict trunk mobility and alter neuromuscular recruitment of core muscles, thereby reducing trunk stability. Specifically, the activation efficiency of core and lower limb muscles is reduced, leading to a delay in postural adjustments (Almurdi, 2024) and a higher risk of falls (Debnath et al., 2024). Furthermore, proprioceptive sensitivity is reduced in obese individuals, weakening their motor perception. Plantar tactile sensitivity is reduced by 46% (Caderby et al., 2020), and a delay in proprioceptive input leads to an extension of postural adjustment reaction time by 200–300 ms (Garcia-Vicencio et al., 2023), which further affects postural stability (Strashko et al., 2022).

A distinctive feature of Chinese martial arts is the integration of internal elements—often described in traditional theory as *Jing* (essence), *Qi* (energy), and *Shen* (spirit)—with external physical movements, a concept that embodies the ancient Chinese cultural philosophy of the unity of form and spirit. The Tai Chi Chuan principle of using the mind to guide *Qi*, and using *Qi* to move the body, along with Hsing-I Chuan's emphasis on three internal and three external harmonies (Wang et al., 2011), are concrete manifestations of a foundational philosophy. Across various Chinese martial arts styles, the intricate fusion of the internal *Jing, Qi*, and *Shen* with external physical forms is essential for achieving a state where the mind moves, the form follows; the form stops, the intent continues. These practices collectively serve as a robust example of the core principles of internal and external cultivation and the unity of form and spirit. From a theoretical perspective, the traditional principles underlying Chinese martial arts—*Jing, Qi*, and *Shen*—can be viewed in modern scientific terms as reflecting physical capacity, psychophysiological regulation, and cognitive-emotional integration. These dimensions correspond to established constructs in sport psychology and motor learning, such as attentional focus, self-regulation, and embodied cognition, which are known to optimize motor performance and skill acquisition (Wulf and Lewthwaite, 2016; Schack and Mechsner, 2006). From a neuroscience perspective, this unity of internal and external elements parallels the coordination of neural mechanisms underlying motor control, interoception, and executive function (Craig, 2009).

Tan Tui, often referred to as the gateway to martial arts, is a foundational Chinese martial arts form. It is characterized by fast and powerful movements, symmetrical patterns, simple postures, ease of learning, and standardized stances (Wu and Wu, 2017). Professor Zhang Wenguang's works on Tan Tui have highlighted its potential benefits for developing strength, speed, and stability (Zhang, 1985). Ancient Chinese medical martial arts texts describe the benefits of Tan Tui as building strength, stabilizing stances, enhancing practicality, and strengthening sinews and bones, indicating its role in enhancing lower-limb strength and balance (Ren, 1985). Our previous research showed that 6 weeks of Tan Tui training with varying loads significantly improved postural control in non-obese college students, with improvements exceeding those observed in Tai Chi Chuan practice under similar conditions (Li et al., 2021). While these findings suggest that Tan Tui may be a particularly efficient modality for balance training, further studies are needed to confirm its relative efficacy.

In this study, we employed electroencephalography (EEG) to explore the neural responses associated with Tan Tui practice in obese college students. We hypothesized that Tan Tui training would be associated with measurable changes in EEG activity relevant to postural control. By examining these effects, we aim to provide evidence on how traditional martial arts exercise may influence neural processes related to motor coordination and balance in obesity. The findings may offer a neurophysiological reference for future exercise-based rehabilitation strategies for obese individuals. The present study proposes the following hypotheses: (1) Traditional martial arts Tan Tui can enhance the absolute power of brain activity in obese college students; (2) Traditional martial arts Tan Tui can improve interhemispheric symmetry in obese college students; (3) Traditional martial arts Tan Tui can enhance brain region synergy in obese college students.

## 2 Materials and methods

### 2.1 Research subjects

The sample size was estimated using G^*^Power 3.1.9 software (Faul et al., 2007). Based on the study's 2 (group) × 2 (time) experimental design, a repeated measures ANOVA was used to test the research hypothesis. Drawing on results from a previous study on the effects of Tan Tui practice on postural control in Tai Chi elective students, the post-training static balance for the experimental group was 111.94 ± 48.34 s, while the control group was 78.16 ± 40.76 s. With an alpha level of 0.05, a power of 0.80, and an allocation ratio of 1, the required sample size was estimated to be 58 participants. To account for potential dropout, while maintaining equal group allocation, the planned total sample size was set at 82 participants (41 per group).

Eighty-two obese college students were recruited from Beijing Sport University. This study was conducted in accordance with the Declaration of Helsinki. The research protocol was reviewed and approved by the Ethics Committee of Beijing Sport University (Approval No. 2021183H). All participants were fully informed of the study procedures and potential risks, and provided written informed consent prior to enrollment. Participants were enrolled if they met the following eligibility criteria: (1) non-physical education majors aged 18–24 years; (2) BMI ≥ 28 kg/m^2^ (Zhao et al., 2022); (3) good physical health with no contraindications for exercise; and (4) voluntary participation with signed informed consent. Potential participants were not eligible if they met any of the following exclusion conditions prior to enrollment: (1) inability to tolerate the exercise intensity of Tan Tui practice; (2) history of injury or surgery within the past 6 months; and (3) pre-existing diseases of the nervous or musculoskeletal systems.

**Figure 1:**
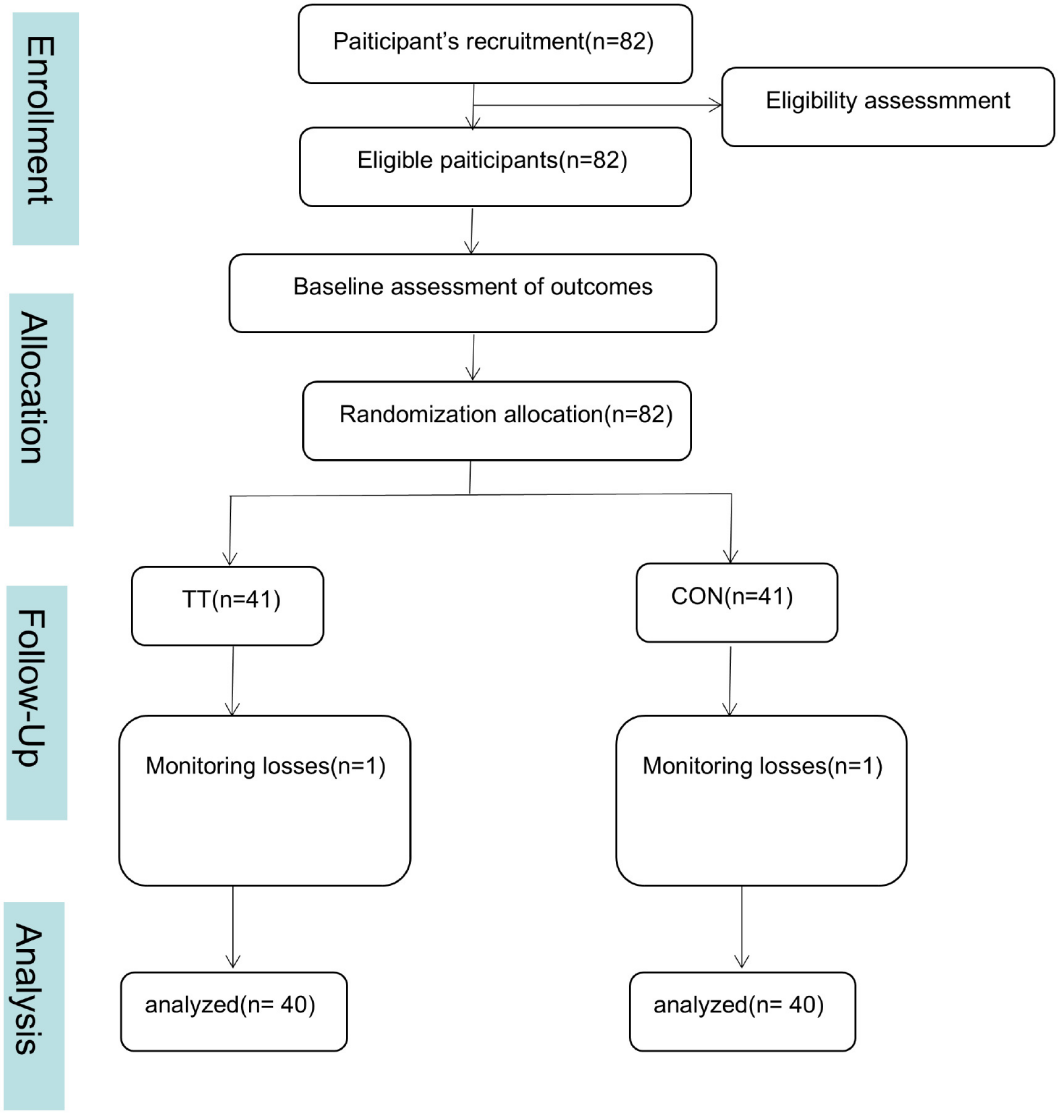
Flow diagram of enrollment subjects.

Participants were randomly allocated to either the Tan Tui practice group (TT, *n* = 41) or the control group (CON, *n* = 41) using a computer-generated randomization sequence conducted by an independent researcher. Figure 1 illustrates the flow diagram of enrollment subjects. During the study period, one participant from the TT group and one participant from the control group were lost to follow-up. Therefore, the final analyzed sample size was 80 participants, consistent across all outcomes, ensuring that the study maintained sufficient statistical power.

As shown in Table 1, an independent samples *t*-test was conducted on participants' age, height, weight, and BMI (Table 1). The results revealed no significant differences (*p* > 0.05) between the groups for all variables, and all data were normally distributed.

**Table 1:** Demographic information statistics of baseline subjects.

**Characteristics**	**Total (*n* = 80)**	**TT (*n* = 40)**	**CON (*n* = 40)**	***p***
Years	18.96 ± 0.47	18.95 ± 0.49	18.96 ± 0.46	0.914
Heights (cm)	174.64 ± 3.62	173.68 ± 3.63	175.61 ± 3.40	0.156
Body weight (kg)	91.71 ± 6.92	90.57 ± 6.82	92.85 ± 6.92	0.136
BMI (kg/m^2^)	31.20 ± 1.690	31.46 ± 1.61	30.93 ± 1.73	0.157

### 2.2 Research methods

#### 2.2.1 Intervention protocol

##### 2.2.1.1 Tan Tui practice

###### 2.2.1.1.1 Selection and standardization of Tan Tui movements

The intervention employed a customized “Two-Road Tan Tui” routine comprising 35 techniques, adapted from Professor Zhang Wenguang's *Ten-Road Tan Tui*. The rationale for movement selection was grounded in three criteria: simplicity of execution, functional practicality, and preservation of traditional characteristics.

**Figure 2:**
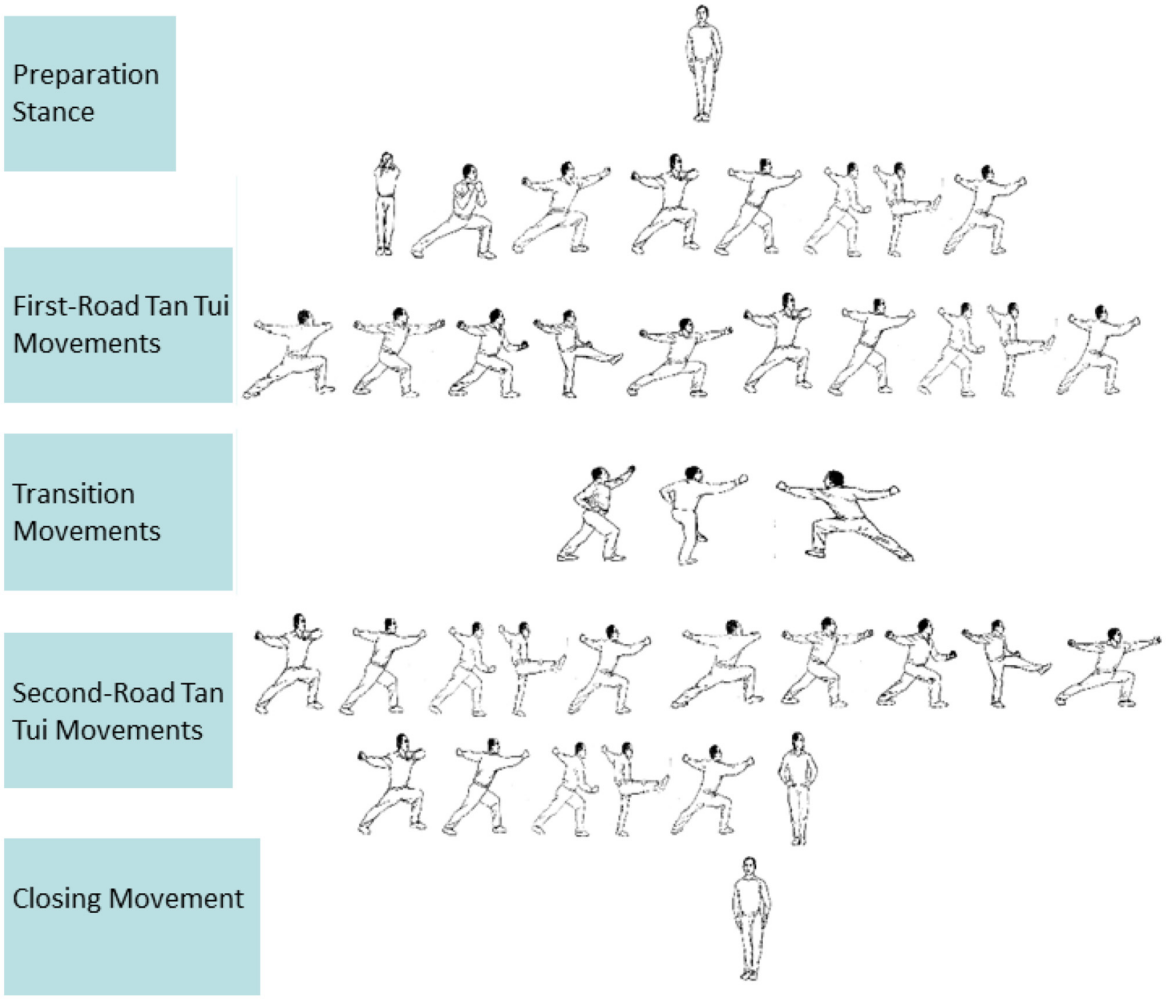
Tan Tui training movement.

The entire movement diagram is shown in Figure 2. To enhance reproducibility, we operationalized the movement set as follows (taking the left stance as an example):

- Preparation stance: standing with feet together.
- First-Road Tan Tui (16 techniques): bow stances, horizontal punches, smashing punches, Tan Tui kicks, and transitions in left-right alternation.
- Transition (3 techniques): palm strike in bow stance, knee lift with punch, directional turn.
- Second-Road Tan Tui (15 techniques): repetition of mirrored combinations with closing movement.
- Closing stance: standing with feet together, palms pressing down.

Technical standards were defined according to the Wushu Taolu Competition Rules (China Martial Arts Association, 2013):

- Range of motion (ROM): For the bow stance, the front thigh was required to be horizontal to the ground, the rear leg straight, and the torso vertical. For the horse stance, both thighs were required to be parallel to the ground with feet forward and torso upright. For the Tan Tui kick, the supporting leg was required to remain straight, the kicking leg lifted to a horizontal position, and the hip angle maintained at approximately 90 °.
- Tempo control: Tempo standardized such that each movement was to be completed with 1.5 s.
- Error tolerance: ±0.25 s was permitted to account for individual variation of tempo. ROM criteria were verified with a goniometer (tolerance ±10 °), and all performances were video-recorded for assessment.

###### 2.2.1.1.2 Intervention protocol

The intervention program lasted 14 weeks, with three sessions per week, each lasting 60 min, comprising a 10-min warm-up, 40 min of Tan Tui practice, and 10 min of cool-down and stretching. A small-group practice method was employed, with groups of three: one participant practicing, one reciting the movements, and one observing. Each participant performed three practice sets per session, each lasting 5 min.

Exercise intensity followed a progressive-load principle, operationalized using heart rate (%HRmax):

- Low intensity: <60% HRmax
- Moderate intensity: 60–80% HRmax
- High intensity: >80% HRmax

Inter-movement pauses of 5, 3, or 1 s were implemented to achieve low, moderate, or high intensity, respectively. Heart rate was continuously monitored with a Polar H10 chest-strap device, and time-in-zone was recorded to verify adherence to target intensity. The weekly progressions are below:

- Weeks 1–4 (Familiarization Phase): Participants learned the full Two-Road Tan Tui set, aiming to complete the routine independently by week 4. Exercise intensity remained at low load (<60% HRmax).
- Weeks 5–6 (Form Refinement Phase): Focused on achieving standardized technical criteria, maintaining low-intensity load.
- Weeks 7–10 (Breathing and Moderate Load Phase): Participants practiced the full set at moderate intensity (60–80% HRmax). Breathing principles were applied: inhale during defensive/gathering movements, exhale during offensive/releasing movements, with coordination of power generation and release.
- Weeks 11–14 (High-Intensity Phase): Practice intensity was progressively increased to high load (>80% HRmax) while maintaining technical standards and breathing coordination.

##### 2.2.1.2 Control group

The control group participated in jogging sessions matching the experimental group in frequency and duration: three sessions per week, 60 min per session, including 10-min warm-up, 40 min of jogging, and 10-min cool-down with stretching.

Jogging intensity followed the same progressive-load principle as the intervention group. Exercise intensity was controlled via target pace and continuous heart rate monitoring using a Polar H10 chest-strap device. Participants were instructed to maintain their heart rate within the prescribed zone, and time-in-zone was recorded for verification. The weekly progressions are:

- Weeks 1–4 (Familiarization Phase): Low-intensity jogging (<60% HRmax) to establish baseline endurance.
- Weeks 5–6 (Form/Technique Phase): Continued low-intensity jogging with emphasis on consistent pace and posture.
- Weeks 7–10 (Moderate Load Phase): Jogging intensity increased to 60–80% HRmax, matching the experimental group's moderate-load Tan Tui practice.
- Weeks 11–14 (High-Intensity Phase): Intensity progressively increased to >80% HRmax, paralleling the experimental group's high-intensity phase.

All participants were able to adhere to the prescribed pace and heart rate targets. No adverse events or injuries occurred during the program.

#### 2.2.2 Testing metrics

**Figure 3:**
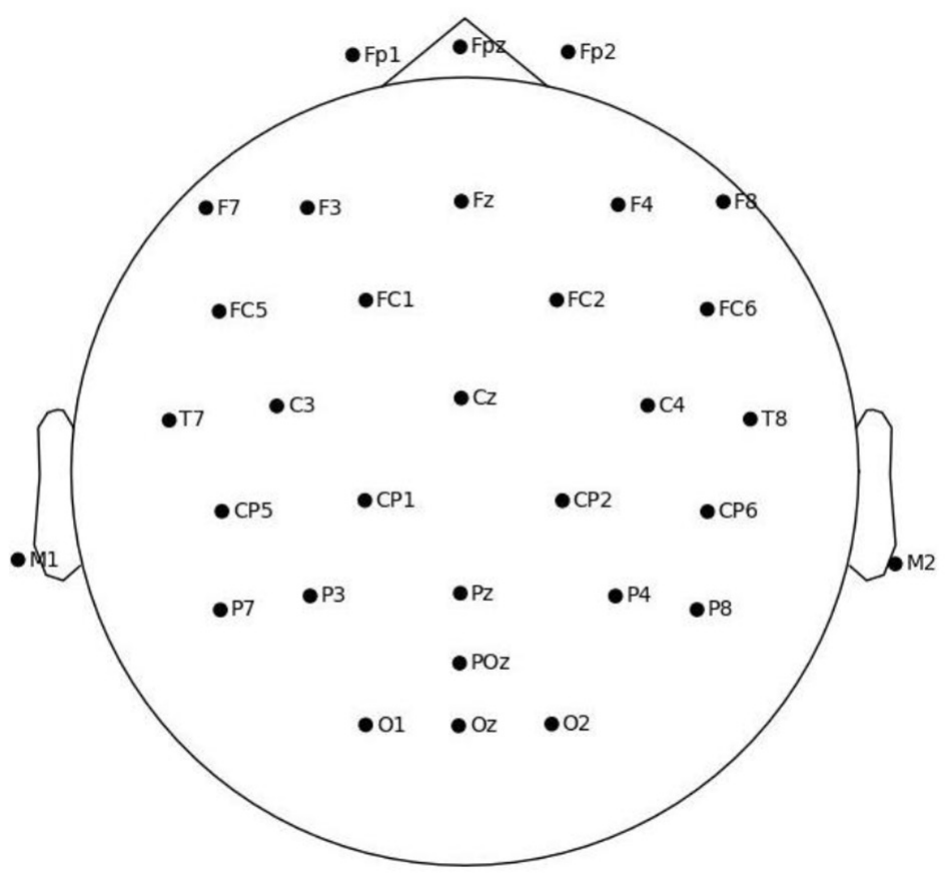
The montage of EEG electrodes.

EEG data were acquired using a 32-channel portable system (EegoTM mylab, Emagine Medical Imaging Solutions GmbH, Germany) with a nano-coated Ag/AgCl electrode cap that required no conductive gel. Signals were recorded according to the international 10–20 system at a sampling rate of 500 Hz, with Cz as the online reference and all electrode impedances kept below 5 kΩ. Artifacts caused by swallowing, eye movements, or muscle activity were marked online and excluded during subsequent preprocessing (Figure 3).

Offline processing was performed using MNE-Python. The continuous data were first filtered with a 0.5 Hz high-pass and a 40 Hz low-pass filter, followed by a 50 Hz notch filter to suppress line noise. Bad channels were interpolated, and artifacts were corrected via Independent Component Analysis (ICA). The data were then re-referenced to the average of the mastoid electrodes.

Power spectral density was estimated using Welch's method with 2-s Hanning windows and 50% overlap. Absolute alpha power (8–13 Hz) with log-transform was computed and averaged across electrodes within each region of interest (ROI). The ROI-electrode mapping was defined in Table 2. We divide the scalp ROIs into a cognitive area (prefrontal, temporal, occipital, parieto-temporal) and a sensorimotor area (posterior frontal, parietal, central). Such a division is consistent with prior EEG research demonstrating that prefrontal, temporal, and occipital regions predominantly subserve higher-order cognitive functions such as executive control, memory, and visual processing, whereas posterior frontal, parietal, and central electrodes reliably capture motor planning, execution, and sensorimotor integration (Başar et al., 1999; Cavanagh et al., 2009). This functional dichotomy provides a principled framework for investigating the differential contributions of cognitive vs. motor networks in martial arts practice.

**Table 2:** ROIs and electrodes included.

**ROI**	**Electrodes**	**Predefined area**
Prefrontal	Fp1, Fp2, Fpz	Cognitive
Posterior-frontal	Fz, F3, F4, F7, F8	Sensorimotor
Central	C3, Cz, C4	Sensorimotor
Temporal	T7, T8	Cognitive
Parietal	Pz, P3, P4	Sensorimotor
Parieto-temporal	P7, P8	Cognitive
Occipital	O1, Oz, O2	Cognitive

To ensure signal quality, participants were instructed to avoid caffeine, and sleep deprivation for 24 h before the experiment. They were asked to arrive with clean and dry hair, and to have consumed a light meal 2–3 h prior to testing. Each session was conducted at a consistent time of day between 9 and 11 a.m. in a sound-attenuated, dimly lit room maintained at 20–24 °C and 20–25% humidity. During the recording, participants sat quietly in a comfortable chair with eyes closed. The resting-state EEG was recorded for 10 min. Mobile phones and other electronic devices were turned off throughout the session.

#### 2.2.3 Statistical analysis

Statistical analysis was performed using the Statistical Package for Social Sciences (SPSS) 25.0. All data were analyzed under the intention-to-treat (ITT) principle. Missing values during the intervention were imputed using multiple imputation by chained equations (MICE). Estimates were pooled following Rubin's rules to account for imputation uncertainty.

**Baseline Data Analysis**. The Shapiro–Wilk test was used to assess normality. Between-group comparability of demographic and baseline variables was examined using independent samples *t*-test.

**Brain Function Data Analysis**. Intervention effects were examined using two-way MANOVA with *Group* (TT vs. CON) and *Time* (pre- vs. post-intervention) as fixed factors, testing for main effects and the *Group* × *Time* interaction. To further account for repeated measures and inter-individual variability, linear mixed-effects models (LMMs) with participants as random intercepts were employed.

**Electrode/ROI Analysis**. For between-group comparisons at each time point, independent-samples *t*-tests were conducted, while within-group pre–post changes were examined using paired-samples *t*-tests. To control for multiple testing across electrodes and ROIs, Bonferroni correction was applied.

**Correlation Analysis**. Relationships between ROIs at different time points were examined using Pearson correlations, conditional on distributional assumptions. Given the exploratory aim of this analysis, no adjustment for multiple comparisons was applied, and results should be interpreted with caution.

A two-tailed *p* < 0.05 was considered statistically significant after adjustment, and *p* < 0.01 was considered highly significant.

## 3 Results

### 3.1 Results of EEG power analysis

In this section, we compared the alpha-band absolute power across electrodes. As shown in Table 3, the repeated-measures ANOVA revealed significant effects of time (*F* = 11.35, *p* < 0.001, η^2^ = 0.799), group (*F* = 5.50, *p* < 0.001, η^2^ = 0.658), and their interaction (*F* = 4.44, *p* < 0.001, η^2^ = 0.609), indicating that temporal dynamics of EEG power differed significantly between the intervention and control groups.

**Table 3:** Multivariate tests of repeated-measures ANOVA on EEG power (*n* = 80).

**Effect**	***F***	***df***	***p***	**η^2^**
Group	5.50	21	<0.001	0.658
Time	11.35	21	<0.001	0.799
Time × Group	4.44	21	<0.001	0.609

For descriptive purposes, Tables 4, 5 present within-group pre–post comparisons for cognitive- and sensorimotor-related ROIs, respectively. In the intervention group, reductions in alpha power were observed at FPz, P7, and O1 (Table 4), as well as F3, P3, P4, and C3 (Table 5) (*p* < 0.05, paired *t*-tests). In contrast, no significant pre–post changes were detected in the control group. These results illustrate that the intervention produced selective within-group changes in alpha power, while the overall between-group differences were captured by the Group × Time interaction in the ANOVA (Table 3).

**Table 4:** Alpha-band (8–13 Hz) absolute power (μV^2^, mean ± SD) in cognitive-related brain regions at rest (eyes-closed).

**Electrode**	**Group**	**Pre**	**Post**	**MD (95% CI)**	***F***	***p***	**η^2^**
FP1	TT	30.88 ± 3.67	30.13 ± 2.28^**^	−0.66 (29.77, 31.23)	1.64	0.204	0.02
	CON	30.5 ± 4.53	31.83 ± 2.34	0.66 (30.44, 31.9)			
FPz	TT	31.21 ± 2.05	29.69 ± 2.37^**^	−1.24 (30.01, 30.89)	15.47	<0.001	0.162
	CON	31.33 ± 2.67	32.05 ± 2.05	1.24 (31.25, 32.14)			
FP2	TT	30.53 ± 2.84	29.93 ± 2.35^**^	−0.58 (29.54, 30.92)	1.39	0.241	0.017
	CON	30.4 ± 3.44	31.23 ± 3.87	0.58 (30.12, 31.51)			
T7	TT	29.42 ± 2.01	28.56 ± 1.98	−0.81 (27.71, 30.27)	0.8	0.373	0.01
	CON	30.26 ± 4.63	29.35 ± 10.11	0.81 (28.53, 31.08)			
T8	TT	29.21 ± 2.4	26.4 ± 0.79^**^	−0.75 (27.23, 28.37)	3.42	0.068	0.041
	CON	28.77 ± 3.37	28.33 ± 2.25	0.75 (27.98, 29.12)			
P7	TT	32.63 ± 2.77	28.4 ± 2.12^**^	−1 (29.84, 31.2)	4.26	0.042	0.051
	CON	32.19 ± 4.37	30.84 ± 3.74	1 (30.84, 32.2)			
P8	TT	31.34 ± 4.48	29.06 ± 3.07	0.28 (29.09, 31.32)	0.13	0.721	0.002
	CON	30.64 ± 5.84	29.19 ± 4.98	−0.28 (28.8, 31.04)			
O1	TT	30.38 ± 4.08	27.45 ± 4.94^*^	−3.05 (27.18, 30.66)	6.09	0.016	0.071
	CON	32.23 ± 4.93	31.71 ± 12.53	3.05 (30.23, 33.71)			
Oz	TT	29.71 ± 4.99	28.51 ± 3.88	−0.37 (27.83, 30.4)	0.17	0.685	0.002
	CON	29.91 ± 5.31	29.06 ± 8.34	0.37 (28.2, 30.77)			
O2	TT	30.04 ± 5.45	28.57 ± 4.72	0.55 (28.27, 30.34)	0.56	0.458	0.007
	CON	29.61 ± 4.11	27.9 ± 5.45	−0.55 (27.72, 29.79)			

Within-group pre–post comparisons are reported as paired *t*-tests.

Values are mean ± SD (μV^2^). Alpha band: 8–13 Hz, eyes-closed resting state. ^*^p < 0.05, ^**^p < 0.01 (within-group significance).

**Table 5:** Alpha-band (8–13 Hz) absolute power (μV^2^, mean ± SD) in sensorimotor-related brain regions at rest (eyes-closed).

**Electrode**	**Group**	**Pre**	**Post**	**MD (95% CI)**	***F***	***p***	**η^2^**
F7	TT	29.46 ± 2.81	24.82 ± 2.73^**^	−0.84 (26.43, 27.85)	2.8	0.098	0.034
	CON	28.74 ± 4.01	27.23 ± 3.31	0.84 (27.28, 28.7)			
F3	TT	28.53 ± 2.52	25.25 ± 2.05^*^	−1.99 (25.63, 28.15)	4.94	0.029	0.058
	CON	29.11 ± 3.55	28.64 ± 10.98	1.99 (27.62, 30.13)			
Fz	TT	28.76 ± 2.34	23.61 ± 5.75^*^	−2.07 (24.6, 27.77)	3.36	0.07	0.04
	CON	28.82 ± 2.14	27.69 ± 12.64	2.07 (26.67, 29.84)			
F4	TT	27.72 ± 2.58	26.47 ± 1.27	−0.81 (26.47, 27.72)	3.33	0.072	0.04
	CON	28.48 ± 4.27	27.32 ± 3.15	0.81 (27.28, 28.52)			
F8	TT	29.24 ± 2.25	26.01 ± 2.86	−1.65 (26.39, 28.87)	3.51	0.065	0.042
	CON	29.72 ± 3.59	28.85 ± 10.31	1.65 (28.04, 30.52)			
P3	TT	28.66 ± 3.69	23.89 ± 3.9^*^	−2.16 (24.78, 27.76)	4.17	0.044	0.05
	CON	28.54 ± 4.6	28.34 ± 10.82	2.16 (26.95, 29.93)			
Pz	TT	25.44 ± 4.03	22.52 ± 5.98	−1.12 (22.36, 25.61)	0.95	0.334	0.012
	CON	25.15 ± 5.22	25.06 ± 11.78	1.12 (23.48, 26.73)			
P4	TT	26.6 ± 5.3	24.2 ± 4.5^*^	−1.58 (24.31, 26.49)	4.2	0.044	0.05
	CON	27.66 ± 5.44	26.3 ± 4.96	1.58 (25.9, 28.07)			
C3	TT	25.83 ± 2.98	21.39 ± 1.51^**^	−3.45 (22.59, 24.63)	22.57	<0.001	0.22
	CON	26.53 ± 4.9	27.58 ± 9.17	3.45 (26.03, 28.08)			
Cz	TT	18.59 ± 4.55	13.32 ± 5.92^*^	−2.15 (14.22, 17.69)	3.03	0.086	0.036
	CON	18.3 ± 5.96	17.9 ± 12.4	2.15 (16.36, 19.84)			
C4	TT	25.35 ± 2.7	22.61 ± 1.55	−0.5 (23.18, 24.78)	0.78	0.38	0.01
	CON	24.92 ± 3.48	24.04 ± 5.37	0.5 (23.68, 25.28)			

Within-group pre–post comparisons are reported as paired *t*-tests.

Values are mean ± SD (μV^2^). Alpha band: 8–13 Hz, eyes-closed resting state. ^*^p < 0.05, ^**^p < 0.01 (within-group significance).

### 3.2 Results of brain region functional symmetry

We further examined hemispheric asymmetry by testing the interaction between Group (TT vs. CON), Time (pre vs. post), and Hemisphere (left vs. right). As shown in Table 6, a repeated-measures ANOVA revealed a significant main effect of Group (*F* = 2.68, *p* = 0.021, η^2^ = 0.177), indicating overall differences in EEG power between intervention and control groups. In contrast, neither the main effect of Time (*F* = 0.22, *p* = 0.968, η^2^ = 0.018) nor the Group × Time interaction (*F* = 0.92, *p* = 0.482, η^2^ = 0.069) reached statistical significance, suggesting no global training-related changes.

**Table 6:** Multivariate tests of repeated-measures ANOVA on EEG power (*n* = 80).

**Effect**	***F***	***df***	***p***	**η^2^**
Group	2.68	75	0.021	0.177
Time	0.22	75	0.968	0.018
Time × Group	0.92	75	0.482	0.069

At the regional level, left–right differences (Δ = Left – Right, mean ± SD) were calculated for prefrontal, temporal, occipital, posterior frontal, parietal, and central regions (Tables 7, 8). Prior to training, no significant hemispheric asymmetry was observed between groups across all regions (*p* = 0.819, 0.901, and 0.92, respectively). Effect sizes (η^2^) were generally small across all regions.

**Table 7:** Hemispheric asymmetry (Δ = Left – Right) in cognitive regions (μV^2^, mean ± SD, *n* = 80).

**Brain area**	**Group**	**Pre Δ (mean ±SD)**	**Post Δ (mean ±SD)**	**MD (95% CI)**	***F***	***p***	**η^2^**
Prefrontal	TT	0.35 ± 4.95	0.19 ± 2.91	0.15 (−1.58, 1.88)	0.053	0.819	0.001
	CON	0.1 ± 6.25	0.60 ± 4.47	−0.5 (−3.05, 2.04)			
Temporal	TT	1.5 ± 6.39	1.50 ± 4.80	−0.01 (−2.57, 2.56)	0.016	0.901	0.000
	CON	3.03 ± 10.22	2.67 ± 11.71	0.36 (−4.78, 5.5)			
Occipital	TT	0.34 ± 6.87	−1.11 ± 6.03	1.45 (−1.26, 4.16)	0.01	0.92	0.000
	CON	2.62 ± 5.91	3.81 ± 13.58	−1.19 (−5.6, 3.21)			

Δ = Left – Right hemispheric power. *p*-values from paired *t*-tests within groups.

**Table 8:** Hemispheric asymmetry (Δ = Left – Right) in sensorimotor regions (μV^2^, mean ± SD, *n* = 80).

**Brain area**	**Group**	**Pre Δ (mean ±SD)**	**Post Δ (mean ±SD)**	**MD (95% CI)**	***F***	***p***	**η^2^**
Posterior frontal	TT	1.03 ± 5.05	−2.41 ± 4.07	3.43 (1.36, 5.51)	1.091	0.299	0.013
	CON	−0.35 ± 7.70	−0.3 ± 17.95	−0.05 (−6.26, 6.17)			
Parietal	TT	2.05 ± 6.47	−0.3 ± 5.95	2.35 (−0.45, 5.15)	0.229	0.634	0.003
	CON	0.87 ± 7.09	2.03 ± 11.29	−1.16 (−5.35, 3.02)			
Central	TT	0.48 ± 4.04	−1.22 ± 2.36	1.7 (0.26, 3.14)	0.01	0.919	0.000
	CON	1.61 ± 5.80	3.54 ± 11.90	−1.94 (−6.41, 2.54)			

Δ = Left – Right hemispheric power. *p*-values from paired *t*-tests within groups.

### 3.3 Evaluation of brain region synergy results

As shown in Table 9, the TT group showed no significant correlations among different cognitive function regions before training (*p* = 0.206, 0.232, and 0.196, respectively). After training, moderate-to-strong positive correlations emerged between the prefrontal–occipital (*r* = 0.524, *p* = 0.040) and temporal–occipital (*r* = 0.71, *p* = 0.003) regions. In the control group, positive correlations were already present before training (all *r* > 0.80, *p* < 0.01), and after training additional associations were observed between the prefrontal–temporal (*r* = 0.436, *p* = 0.048) and temporal–occipital (*r* = 0.812, *p* < 0.001) regions. As shown in Table 10, both groups exhibited positive correlations among sensorimotor regions both before and after training (all *r* > 0.65, *p* < 0.01).

**Table 9:** Correlation of power among different cognitive function regions within groups, pre- and post-training.

**Brain area**	**Group**	***r* before training**	***p***	***r* after training**	***p***
Prefrontal-temporal	TT	0.323	0.206	0.573	0.032
	CON	0.823	0.002	0.436	0.048
Prefrontal-occipital	TT	0.352	0.232	0.524	0.040
	CON	0.826	<0.001	0.284	0.326
Temporal-occipital	TT	0.402	0.196	0.713	0.003
	CON	0886	<0.001	0.812	<0.001

**Table 10:** Correlation of power among different sensorimotor function regions within groups, pre- and post-training.

**Brain area**	**Group**	***r* before training**	***p***	***r* after training**	***p***
Posterior frontal-parietal	TT	0.821	<0.001	0.693	0.010
	CON	0.726	<0.001	0.816	0.002
Posterior frontal-central	TT	0.861	<0.001	0.887	<0.001
	CON	0.653	0.003	0.746	<0.001
Parietal-central	TT	0.916	<0.001	0.896	<0.001
	CON	0.842	<0.001	0.851	0.003

As shown in Table 11, exploratory Pearson correlation analyses were conducted between sensorimotor regions (posterior frontal, parietal, central) and cognitive function–related regions (prefrontal, temporal, occipital).

**Table 11:** Pearson correlation of power between cognitive and sensorimotor function regions within groups, pre- and post-training.

**Conditions**	**Brain area**	**Prefrontal *r***	***p***	**Temporal *r***	***p***	**Occipital *r***	***p***
TT before training	Posterior frontal	0.654	0.005	0.518	0.041	0.582	0.031
	Parietal	0.241	0.326	0.336	0.193	0.936	<0.001
	Central	0.335	0.138	0.643	0.008	0.759	<0.001
CON before training	Posterior frontal	0.718	0.002	0.839	<0.001	0.756	<0.001
	Parietal	0.316	0.286	0.653	0.007	0.845	<0.001
	Central	0.334	0.458	0.483	0.071	0.648	0.016
TT before training	Posterior frontal	0.563	0.020	0.608	0.012	0.712	0.008
	Parietal	−0.661	0.009	0.683	0.004	0.942	<0.001
	Central	−0.824	<0.001	0.529	0.060	0.635	<0.001
CON after training	Posterior frontal	−0.082	0.816	0.482	<0.001	0.534	0.038
	Parietal	0.271	0.305	0.641	0.007	0.759	0.001
	Central	−0.163	0.562	0.493	0.056	0.746	<0.001

In the TT group, prior to training, several correlations were observed between posterior-frontal and cognitive regions, parietal and occipital regions, as well as central and temporal/occipital regions (r range = −0.82 to 0.78, *p* < 0.05). These included both positive and negative associations. Following training, correlations appeared more widespread, involving nearly all sensorimotor–cognitive region pairs (r range = −0.71 to 0.87, *p* < 0.05).

In the control group, pre-training patterns were broadly similar, with correlations detected among posterior-frontal and cognitive regions, parietal and occipital regions, and central and temporal/occipital regions (r range = −0.78 to 0.83, *p* < 0.05). After training, correlations were primarily observed between posterior-frontal and temporal/occipital regions, parietal and temporal/occipital regions, and central and occipital regions (r range = −0.65 to 0.81, *p* < 0.05).

Given that no correction for multiple comparisons was applied, these findings should be regarded as preliminary and interpreted with caution.

## 4 Discussions

In this study, we explored the neural effects of Tan Tui practice in obese college students using EEG. Our findings provide preliminary evidence that this traditional martial arts exercise may modulate brain activity, enhance interhemispheric symmetry, and promote functional coordination among brain regions relevant to motor control. These results offer an initial neurophysiological basis for considering Tan Tui as a potential intervention to support motor function and balance in obese individuals, while acknowledging the exploratory nature of the study.

### 4.1 The impact of traditional martial arts Tan Tui practice on EEG power

The results of this study show that after 14 weeks of Tan Tui practice, the experimental group exhibited a significant decrease (*F* = 4.44, *p* < 0.001, η^2^ = 0.609) in the absolute power of the alpha band across both cognitive (prefrontal, temporal, occipital) and sensorimotor (posterior frontal, parietal, central) brain regions during a resting-eyes-closed state. Prior studies have consistently linked alpha power reduction—often interpreted as “alpha desynchronization”—to increased cortical excitability and enhanced readiness for information processing (Klimesch, 2012; Jensen and Mazaheri, 2010). Importantly, such desynchronization is not simply an increase in “activity,” but rather reflects the functional reorganization of neural assemblies in preparation for motor or cognitive demands. This aligns with the observed effect sizes in our study, which indicate a moderate-to-strong training effect in key regions involved in postural control.

In contrast, the control group (jogging) showed no significant changes in alpha power after training. This supports the idea that conventional aerobic exercise may not substantially alter neural patterns that underlie complex motor control, which are considered relatively “costly” to reorganize. By contrast, Tan Tui practice significantly reduced alpha power, suggesting that this traditional martial arts training may facilitate neural efficiency—defined as the ability to achieve greater performance with lower or more optimized neural activation (Neubauer and Fink, 2009). This interpretation is reinforced by the consistency of our results with prior motor learning studies, which demonstrate that alpha desynchronization is a robust marker of neural plasticity during skill acquisition and error correction (Ortiz et al., 2023).

Taken together, these findings indicate that Tan Tui training induces functional remodeling of cortical networks in obese college students, beyond the effects of general aerobic activity. The reduction in alpha power, accompanied by moderate-to-large effect sizes, provides converging evidence that this practice enhances the brain's ability to maintain a more “ready” state at rest, thereby enabling more efficient recruitment of neural resources when engaging in postural control tasks.

### 4.2 The impact of traditional martial arts Tan Tui practice on brain region symmetry

Our study found that Tan Tui practice improved the left-right hemispheric symmetry in TT group (repeated-measures ANOVA revealed a significant main effect of Group with *F* = 2.68, *p* = 0.021, η^2^ = 0.177). This indicates that functional symmetry of motor-related cortices—a crucial neural foundation for coordinated bilateral limb movements and midline stability—was enhanced following training.

Obesity is known to induce uneven body load distribution and altered gait, thereby exacerbating asymmetry in muscle strength and neural control between the two sides of the body (Błaszczyk et al., 2009; Sharma et al., 2024). The design of Tan Tui routines emphasizes “left-right symmetry and reciprocal practice,” requiring equal engagement of both sides of the body (e.g., left vs. right bow stance punches and kicks). Such systematic bilateral training likely facilitates parallel recruitment of the left and right hemispheres during motor planning and execution, strengthening interhemispheric neural connectivity (Yalfani and Raeisi, 2022). Consistent with this interpretation, similar effects have been reported in Taiji and dance, which enhance interhemispheric coordination and motor cortex integration (Chen et al., 2022; Wind et al., 2020).

Importantly, the improvement observed in the central lobe (C3/C4, corresponding to the primary motor cortex, M1) was statistically greater in the Tan Tui group compared to the jogging group suggesting that the benefits were not simply attributable to bilateral movement *per se*. Jogging, although involving both limbs, is highly automated and imposes fewer demands on precise interhemispheric coordination, which explains the absence of significant improvements in symmetry. In contrast, Tan Tui's symmetrical practice paradigm effectively reshaped the functional balance of the motor cortex in obese college students, representing a key neural mechanism for improving postural control.

### 4.3 The impact of traditional martial arts Tan Tui practice on brain region synergy

Our results reveal that after training, the experimental group showed enhanced correlations among cognitive regions (e.g., prefrontal–occipital, temporal–occipital) and between cognitive and sensorimotor regions (posterior frontal, parietal, central, and various cognitive areas). Specifically, these functional associations showed moderate-to-strong effect sizes (e.g., posterior-frontal and central *r* = 0.8611, *p* < 0.001), suggesting that Tan Tui practice may support more integrated neural communication to meet the complex demands of postural control. While these findings are correlational in nature and were not corrected for multiple comparisons, the consistency across several key networks increases confidence that the effects reflect a genuine trend rather than chance.

Traditional Chinese martial arts emphasize the “unity of internal and external, form and spirit,” which can be framed as the integration of cognitive and sensorimotor processes. Similar interpretations have been proposed in studies of mind–body practices such as yoga, which demonstrate strengthened prefrontal–sensorimotor and multisensory connectivity following training (Villemure et al., 2015). Tan Tui practice similarly requires continuous attention (prefrontal), proprioceptive monitoring (parietal), precise motor execution (central), and multimodal integration (temporal, occipital), thereby providing an ecological platform for the brain to enhance large-scale network cooperation.

In obese individuals, previous work has shown altered functional connectivity patterns, including hyper-connectivity in reward networks (Park et al., 2020) and abnormal synchronization in low-frequency bands (Olde Dubbelink et al., 2008), which may contribute to inefficient cognitive–motor integration. Our results suggest that Tan Tui practice may partially counteract these patterns by promoting long-range functional associations. For example, strengthened prefrontal–sensorimotor correlations indicate stronger top-down control of motor planning, whereas enhanced parietal–occipital–temporal links suggest better multisensory integration. Although further studies with stricter statistical controls (e.g., multiple-comparison correction, graph-theoretic network analysis) are needed, the present findings provide preliminary evidence that Tan Tui practice induces functional reorganization at the whole-brain network level, distinguishing it from more automated exercises such as jogging.

### 4.4 Limitations

The analyses in this study were conducted in an exploratory manner to identify potential relationships and differences among predefined cognitive and sensorimotor regions of interest (ROIs). Given the limited number of a priori ROIs and the relatively small sample size, uncorrected *p*-values in correlation analysis were reported to avoid overly conservative adjustments that could obscure meaningful patterns. Nevertheless, these results should be interpreted with caution, and future studies with larger samples and confirmatory designs are warranted to validate these findings with appropriate multiple comparison corrections.

While the control group engaged in jogging matched the intervention group in terms of frequency, duration, and relative exercise intensity (%HRmax), it differed from the Two-Road Tan Tui practice in cognitive-motor complexity. Tan Tui involves coordinated sequences of postures, strikes, kicks, and breathing patterns, requiring continuous motor planning, balance control, and integration of attentional and executive functions. In contrast, jogging primarily constitutes repetitive, rhythmic locomotor activity with minimal cognitive demand.

This difference in movement complexity and cognitive engagement may have contributed to additional neuromuscular and postural benefits in the intervention group, independent of cardiovascular load. Therefore, while the groups were matched for aerobic intensity and total exercise volume, the disparity in motor-cognitive demands represents a potential limitation of the study and should be considered when interpreting the results.

## 5 Conclusions

This study used traditional martial arts Tan Tui practice as an intervention to explore its effects on the brain mechanisms of postural control in obese college students, using EEG technology. The results show that after 14 weeks of systematic Tan Tui training, the experimental group exhibit enhanced absolute power, improved interhemispheric symmetry, and increased brain region synergy in obese college students. In summary, traditional martial arts Tan Tui practice can serve as an effective exercise intervention for obese adolescents to improve postural control and functional brain imbalances, providing a theoretically and practically distinct pathway rooted in Chinese culture for exercise rehabilitation and promoting brain health.

## Data Availability

The data generated and/or analyzed during the current study are available from the corresponding author upon reasonable request.
